# Implications of Ecological Niche Differentiation in Marine Bacteria for Microbial Management in Aquaculture to Prevent Bacterial Disease

**DOI:** 10.1371/journal.ppat.1005843

**Published:** 2016-11-10

**Authors:** Tom Defoirdt

**Affiliations:** 1 Center for Microbial Ecology and Technology (cmet), Ghent University, Ghent, Belgium; 2 Laboratory of Aquaculture & *Artemia* Reference Center, Ghent University, Ghent, Belgium; The University of North Carolina at Chapel Hill, UNITED STATES

Aquaculture, breeding, and rearing of aquatic animals (mollusks, crustaceans, and finfish) in marine, brackish, and freshwater bodies is playing an increasingly important role with respect to food security for the growing human population and is predicted to dominate the seafood supply within a few decades [1]. However, the further sustainable expansion of the sector is currently hampered by a number of factors, amongst which diseases are playing a prominent role, especially in the early life stages of the animals (i.e., larviculture [2]). A major group of causative agents are bacterial pathogens, such as *Vibrio* spp. [3]. These bacteria cause huge losses in the aquaculture industry worldwide, with acute hepatopancreatic necrosis disease (AHPND) as a notable recent example [4]. This disease, caused by strains of *Vibrio parahaemolyticus* that acquired a plasmid encoding two toxin genes, was first reported in southern China in 2009,subsequently spread over Southeast Asia, and reached Mexico in 2013. The AHPND disease typically affects shrimp postlarvae, within 20–30 days after stocking, and frequently causes up to 100% mortality. Global losses in the shrimp farming industry because of this disease have been estimated to be over US$1,000 million per year [5]. Pathogenic vibrios are opportunistic pathogens (as opposed to obligate pathogens) since they are capable of surviving and multiplying in the absence of their host [6].

In a recent paper, De Schryver and Vadstein argued that the ecological r/K theory could serve as a foundation for the development of microbial management strategies to prevent diseases caused by opportunistic pathogens in aquaculture [7]. According to the ecological theory of r/K selection, an unstable environment containing high nutrient levels per individual selects for organisms with the capacity to exploit nutrients and increase population size, termed r-strategists. On the other hand, a stable environment where the resources per individual are scarce will select for slow-growing organisms, termed K- strategists. Most bacterial diseases in aquaculture (and especially larviculture) are caused by opportunistic pathogens that are ubiquitous in the marine environment and that are capable of quickly increasing their population size in the aquaculture environment: i.e., r-strategists [2,7]. The triggers that induce mortality events are not yet completely understood (and are probably different for different pathogens), although increases in dissolved nutrients, temperature, and periods of hypoxia have been shown to be involved [8–10].

Management strategies aiming at disease prevention in aquaculture systems should be pursued at different levels, the first of which includes the implementation of hygienic barriers (e.g., the disinfection of incoming water in order to avoid those pathogens that are part of the normal marine microbiota from entering the system). These hygienic barriers are, however, not flawless and do not result in a complete eradication of all incoming bacteria, and consequently, additional measures should be taken to restrain pathogens within the system [11]. Moreover, disinfection practices can favor r-strategists because they decrease the competition between bacteria (by decreasing the bacterial density), and this also holds for feeding regimes that result in large fluctuations in nutrient levels in the rearing water [12]. Given the fact that many of the major bacterial aquaculture pathogens are r-strategists, theoretically, by imposing slow growth conditions in order to favor K-strategists (i.e., organisms capable of thriving in an environment with low levels of nutrients per individual), the pathogen pressure in the aquaculture system can be decreased, and this should result in a lower incidence of diseases [7]. Slow growth conditions can be imposed by controlled microbial colonization of the (disinfected) inflow water with K-strategists (matured water [13]) and by avoiding large fluctuations in nutrient levels in the water—e.g., by imposing a feeding regime consisting of a continuous administration of low feed doses instead of a regime consisting of few feeding events in which relatively large amounts of feed are introduced in the system. The reasoning behind this is that dissolved nutrient levels will be kept low because of consumption by the microbiota in the matured water, thereby minimising opportunities for opportunistic r-strategists to invade the system. This approach has been experimentally validated (e.g., in Atlantic cod larval rearing, in which a matured water approach resulted in an increased survival [14]). However, the success seems to be variable, and survival rates in systems with matured water are often still relatively low (although higher than in other systems). Hence, although microbial management based on slow growth to favor K-strategists is intellectually appealing and to some extent supported by experimental data, there still seems to be room for improvement. In the following paragraphs, I will argue that microbial management strategies need to take into account the fact that nutrients are not homogeneously distributed in the water column. Indeed, recent findings with respect to ecological niche differentiation of marine bacteria indicate that there still is an open window for invasion by opportunistic pathogens in matured water that has been colonised by K-strategists because these pathogens have evolved mechanisms to find and exploit hot spots with high nutrient levels [15,16].

Despite its superficial homogeneous appearance, the marine water column can have a diverse physical, chemical, and biological microenvironment, and nutrients are not homogeneously distributed at scales relevant to microorganisms but rather occur as hot spots, as they are, e.g., often associated with or released from particles such as microalgae, fecal pellets, and marine snow [16]. In order to cope with the conditions that prevail in the marine environment (i.e., low nutrient levels in the bulk and high levels in hot spots), marine bacteria have evolved two divergent strategies: they are either (1) minute and nonmotile, with a streamlined genome, or (2) relatively large and motile, with a high metabolic flexibility [16]. The small cell size of the first group allows them to maximise uptake per unit of biomass and to obtain nutrients at the low bulk concentrations of the oceans [17]. Their streamlined genomes imply poor metabolic plasticity and an inability to exploit high-resource conditions that occur in hot spots [18]. The second, motile group is adapted to exploit these relatively rare, resource-rich conditions. Chemotactic motility enables them to access novel, nutrient-rich hot spots [16]. Their metabolic flexibility allows them to adapt rapidly to newly encountered microenvironments [19]. Interestingly, the division between both strategies described above is broadly aligned with the dichotomy between K-strategists and r-strategists, and the abundance of r-strategists will be a reflection of the patchiness of the water column [16]. The Vibrionaceae family contains the major bacterial pathogens in marine aquaculture, and they belong to the second group: they are often (highly) motile, show a high metabolic flexibility, and are capable of quickly increasing their population size in the environment if conditions are favourable [16].

Similar to oceans, the water column of aquaculture systems is not homogeneous, and nutrients are usually also present in hot spots (fecal pellets, uneaten feed particles), which are highly abundant in the rearing water of an aquaculture system (at least when compared to the presence of hot spots in the ocean). Hence, there are plenty of opportunities for r-strategists (including opportunistic pathogens) in aquaculture systems. If nutrients were uniformly distributed, then matured water (containing a high level of K-strategists) would adequately limit the risk of invasion by opportunistic pathogens (Fig 1A). However, the fact that nutrients are not uniformly distributed includes a significant risk for invasion by opportunistic pathogens, even in matured water, because they have a competitive advantage over K-strategists with respect to obtaining nutrients from hot spots (and thereby increasing their population density) (Fig 1B). Along this line, Lemire et al. recently reported that oyster pathogenic vibrios belong to distinct ecological populations that show preference for zooplankton and large particles [20].

**Fig 1:**
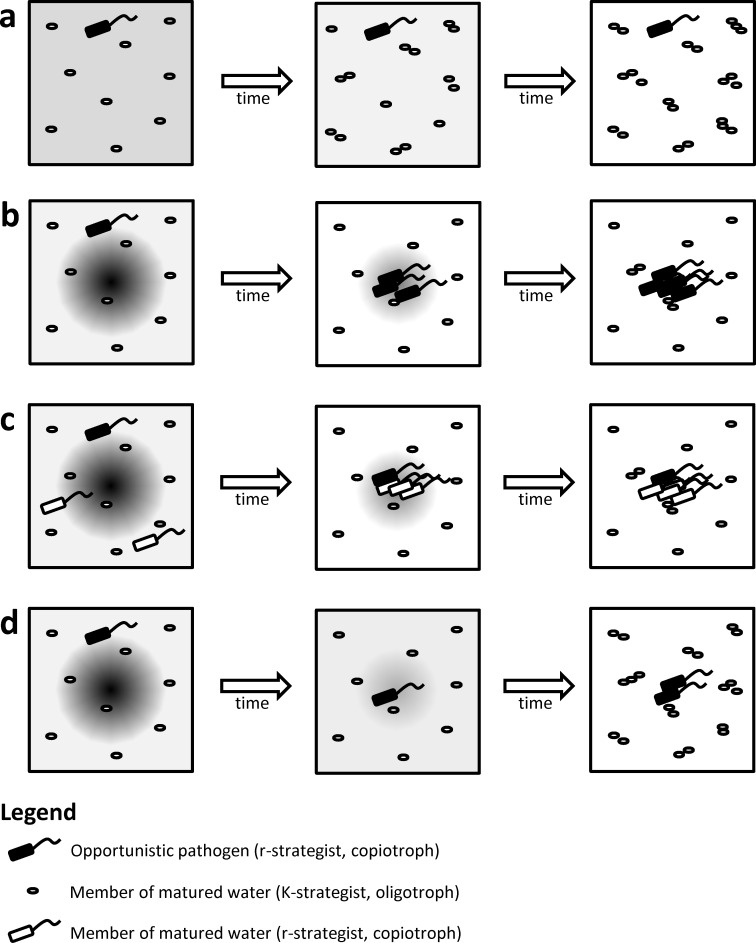
Schematic representation of the impact of nutrient distribution on the opportunities for an opportunistic pathogen to invade the preexisting microbial community in an aquaculture system (matured water). The darker the colour, the higher the nutrient concentration. **(A)** In the hypothetical scenario in which nutrients are homogeneously distributed in the water column, K-strategists are capable of using the nutrients, and there is no opportunity for the opportunistic pathogen to reach a high population density. **(B)** In reality, nutrients are not homogeneously distributed but rather present as hot spots with locally high nutrient concentrations. The nonmotile K-strategists remain randomly distributed and are not able to adequately exploit the nutrient source. Chemotactic motility enables the opportunistic pathogen to localise and exploit the hot spot, and as a consequence, its population density increases considerably. **(C)** If the original microbial community of the matured water also contains r-strategists, then these r-strategists can compete with opportunistic pathogens for the resources present in the hot spots. As a consequence, the opportunistic pathogen is not able to reach a density as high as in panel **B**. **(D)** If feed particles are used that disintegrate rapidly, then the time window during which the hot spot exists is limited and nutrients quickly diffuse into the bulk, thereby avoiding (locally) high levels of dissolved nutrients. As a consequence, K-strategists will be capable of utilising a significant fraction of the nutrients, and the opportunistic pathogen is not able to reach a density as high as in panel **B**.

Although ecological niche differentiation in marine bacteria includes a risk for invasion of aquaculture systems by opportunistic pathogens, further research is needed in order to fully appreciate the magnitude of this risk and to develop novel microbial management strategies to limit the risk of invasion by opportunistic pathogens in an aquaculture system. Such a strategy could consist of including nonpathogenic r-strategists in the matured water (Fig 1C). Indeed, in this way, the rearing water would also contain bacteria that are able to occupy the ecological niche that is prone to invasion by opportunistic pathogens. It is clear that in this case, r-strategists should be carefully selected—e.g., taking into account the fact that within the Vibrionaceae, ecological population boundaries can be at a low phylogenetic level (e.g., several ecologically distinct populations could be distinguished within the species *V*. *splendidus* [21]). On the other hand, recent work indicates that a large fraction of strains within a certain ecological population (although not all of them) can be pathogenic [21], and consequently, it might prove to be challenging (though not impossible) to identify appropriate r-strategists that could be used as inoculum for the precolonization of the intake water. An additional strategy is to use feed pellets that disintegrate relatively quickly, as this will decrease the window during which r-strategists have an advantage over the K-strategists present in the matured water (Fig 1D). Indeed, if the pellets disintegrate quickly, then the time frame during which local dissolved nutrient levels are high will be limited, as the nutrients will diffuse more quickly into the bulk water, where they can be consumed by K-strategists. It needs to be stressed, however, that these pellets should be used in combination with a slow feeding regime (see above) in order to avoid temporarily high levels of dissolved nutrients in the water.

## References

[1] World Bank (2013) Fish to 2030. Prospects for Fisheries and Aquaculture. World Bank Report n° 83177-GLB. The World Bank, Washington DC. 80 pp.

[2] Vadstein O, Bergh O, Gatesoupe FJ, Galindo-Villegas J, Mulero V, et al (2013) Microbiology and immunology of fish larvae. Rev Aquacult 5: S1–S25

[3] Defoirdt T, Sorgeloos P, Bossier P. (2011) Alternatives to antibiotics for the control of bacterial disease in aquaculture. Curr Opin Microbiol 14: 251–258. 10.1016/j.mib.2011.03.004 21489864

[4] Lee CT, Chen IT, Yang YT, Ko TP, Huang YT, et al (2015) The opportunistic marine pathogen *Vibrio parahaemolyticus* becomes virulent by acquiring a plasmid that expresses a deadly toxin. Proc natl Acad Sci USA 112: 10798–10803. 10.1073/pnas.1503129112 26261348 PMC4553777

[5] FAO Fisheries and Aquaculture. (2013) Report of the FAO/MARD technical workshop on early mortality syndrome (EMS) or acute hepatopancreatic necrosis syndrome (AHPNS) of cultured shrimp. Report n° 1053. Hanoi, Vietnam.

[6] Brown SP, Cornforth DM, Mideo N. (2012) Evolution of virulence in opportunistic pathogens: generalism, plasticity, and control. Trends Microbiol 20: 336–342. 10.1016/j.tim.2012.04.005 22564248 PMC3491314

[7] De Schryver P, Vadstein O. (2014) Ecological theory as a foundation to control pathogenic invasion in aquaculture. ISME J 8: 2360–2368. 10.1038/ismej.2014.84 24892581 PMC4260705

[8] Kimes NE, Grim CJ, Johnson WR, Hasan NA, Tall BD, et al (2012) Temperature regulation of virulence factors in the pathogen *Vibrio coraliilyticus* . ISME J 6: 835–846. 10.1038/ismej.2011.154 22158392 PMC3309362

[9] Eggermont M, Tamanji A, Nevejan N, Bossier P, Sorgeloos P, et al (2014) Stimulation of heterotrophic bacteria associated with wild-caught blue mussel (*Mytilus edulis*) adults results in mass mortality. Aquaculture 431: 136–138.

[10] Phippen BL, Ivanina AV, Sokolova IM, Oliver JD. (2016) *Vibrio coraliilyticus* causes rapid immune response and mortality in the eastern oyster, *Crassostrea virginica*, after exposure to anoxia In: Le Roux F. (ed). Abstracts of Vibrio 2016, Roscoff, France.

[11] De Schryver P, Defoirdt T, Sorgeloos P. (2014) Early mortality syndrome outbreaks: a microbial management issue in shrimp farming? PLOS Path 10: e1003919. 10.1371/journal.ppat.1003919 PMC3999206 24763380

[12] Blancheton JP, Attramadal KJK, Michaud L, Roque d’Orbcastel E, et al (2013) Insight into bacterial population in aquaculture systems and its implication. Aquacult Eng 53: 30–39.

[13] Skjermo J, Salvesen I, Øie G, Olsen Y, Vadstein O. (1997) Microbially matured water: a technique for selection of a non-opportunistic bacterial flora in water that may improve performance of marine larvae. Aquacult Int 5: 13–28.

[14] Attramadal KJK, Truong TMH, Bakke I, Skjermo J, Olsen Y, et al (2014) RAS and microbial maturation as tools for K-selection of microbial communities improve survival in cod larvae. Aquaculture 432: 483–490.

[15] Grossart HP. (2010) Ecological consequences of bacterioplankton lifestyles: changes in concepts are needed. Environ Microbiol Rep 2: 706–714. 10.1111/j.1758-2229.2010.00179.x 23766274

[16] Stocker R. (2012) Marine microbes see a sea of gradients. Science 338: 628–633. 10.1126/science.1208929 23118182

[17] Lauro FM, McDougald D, Thomas T, Williams TJ, Egan S, et al (2009) The genomic basis of trophic strategy in marine bacteria. Proc Natl Acad Sci USA 106: 15527–15533. 10.1073/pnas.0903507106 19805210 PMC2739866

[18] Yooseph S, Nealson KH, Rusch DB, McCrow JP, Dupont CL, et al (2010) Genomic and functional adaptation in surface ocean planktonic prokaryotes. Nature 468: 60 10.1038/nature09530 21048761

[19] Ayo B, Unanue M, Azua I, Gorsky G, Turley C, et al (2001) Kinetics of glucose and amino acid uptake by attached and free-living marine bacteria in oligotrophic waters. Mar Biol 138: 1071–1076.

[20] Lemire A, Goudenège D, Versigny T, Petton B, Calteau A, et al (2015) Populations, not clones, are the unit of vibrio pathogenesis in naturally infected oysters. ISME J 9: 1523–1531. 10.1038/ismej.2014.233 25489729 PMC4478691

[21] Hunt DE, David LA, Gevers D, Preheim SP, Alm EJ, et al (2008) Resource partitioning and sympatric differentiation among closely related bacterioplankton. Science 320: 1081–1085. 10.1126/science.1157890 18497299

